# Using a Health Informatics System to Assess Effect of a Federal Cigarette Tax Increase on Readiness to Quit Among Low-Income Smokers, Louisiana, 2009

**DOI:** 10.5888/pcd11.130203

**Published:** 2014-04-03

**Authors:** Tung-Sung Tseng, Sarah Moody-Thomas, Ronald Horswell, Yong Yi, Michael D. Celestin, Krysten D. Jones

**Affiliations:** Author Affiliations: Sarah Moody-Thomas, Ronald Horswell, Yong Yi, Michael D. Celestin, Krysten D. Jones, Louisiana State University, New Orleans, Louisiana.

## Abstract

**Introduction:**

Health informatics systems are a proven tool for tobacco control interventions. To address the needs of low-income groups, the Tobacco Control Initiative was established in partnership with the Louisiana State University Health Care Services Division to provide cost-effective tobacco use cessation services through the health informatics system in the state public hospital system.

**Methods:**

In this study we used a Web-based, result-reporting application to monitor and assess the effect of the 2009 federal cigarette tax increase. We assessed readiness to quit tobacco use before and after a cigarette tax increase among low-income tobacco users who were outpatients in a public hospital system.

**Results:**

Overall, there was an increase in readiness to quit, from 22% during the first week of February to 33% during the first week of April, when the tax went into effect. Smokers who were female, 31 or older, African American, and assessed at a clinic visit in April were more likely to report readiness to quit than were men, those aged 30 or younger, those who were white, and those who were assessed at a clinic visit in February.

**Conclusion:**

A health informatics system that efficiently tracks trends in readiness to quit can be used in combination with other strategies and thus optimize efforts to control tobacco use. Our data suggest that a cigarette tax increase affects smokers’ readiness to quit and provides an opportunity to intervene at the most beneficial time.

## Introduction

Tobacco use, a long-standing public health problem, remains the leading cause of preventable illness and death (1). Each year, an estimated 443,000 people in the United States die of a smoking-related disease, based on 2000 through 2004 data (2). Tobacco use is implicated in 85% of lung cancer cases and one-third of all cancer deaths (2,3).

The average price that Louisiana smokers reported paying for their last pack of cigarettes was $4.61 in 2009 and 2010; the range among states was $4.04 to $7.98. The average price that Louisiana smokers reported paying for their last carton of cigarettes was $36.38 in 2009 and 2010; the range among 45 states with valid data was $30.46 to $64.45 (2). Although the federal excise tax increased in 2009 from 39 cents to $1.01 per pack, Louisiana has the third lowest state cigarette tax price per pack in the United States ($0.36) (2). Raising the price of cigarettes affects tobacco use across a range of racial, socioeconomic, and age groups. Price increases deter initiation, decrease consumption, and lower prevalence of tobacco use (2,4). When the cost of tobacco products rises, adult smokers are more likely to make an attempt to quit (4). In addition, younger smokers are more sensitive to price than adult smokers (5,6). However, the effect of rising prices of tobacco products on young people is more the result of preventing initiation rather than cessation (7). Lower-income smokers are especially responsive to price increases; they are more likely to reduce consumption or quit than higher-income smokers (8). A 10% increase in the price of tobacco products leads to a 2% to 5% decrease in consumption of cigarettes (7).

Promotional and outreach strategies implemented in anticipation of the $0.61 federal tax increase, which took effect April 1, 2009, helped to double the volume of calls to the national quitline (9). Thus, the interval preceding a price increase offers an opportunity to promote cessation services. Because most measures of the effect of a tobacco tax increase have been made after the tax has taken effect, there is limited understanding of how a pending tax affects smokers’ progression toward making an attempt to quit. Because an increase in tax on tobacco products is publicized before the date it goes into effect, the “anticipatory phase” offers an opportunity to maximize the effect of the tax. Thus, it is important to understand predictors of cessation and how they might be used in combination with other strategies to increase quitting rates. In addition, understanding the readiness to quit, quit attempts, and associated factors may assist health care providers to deliver more effective nicotine-dependence treatment to their patients (10). Readiness to quit and previous attempts to quit predict successful quitting (11,12).

Health informatics systems and a public health–oriented electronic health record (EHR) (13,14) are tools that can be used in concert with tobacco control interventions to identify the time at which to interview smokers and document the effect of intervention, including tax increases. Boyle et al reviewed 11 studies published between January 1990 and May 2011 and found modest improvements in some of the recommended clinician action steps on tobacco use (15). However, documentation of tobacco use status and referral to cessation counseling were increased by using the EHR to record and treat patient tobacco use at medical visits (15). EHRs have demonstrated efficiency as part of an integrated approach to systems changes to support tobacco use intervention (14,16). We believe the Louisiana public hospital system is among the first public hospital EHR systems using the 5 A’s (Ask, Advise, Assess, Assist, and Arrange) from the Clinical Practice Guideline of the US Public Health Service (17) to monitor patients’ tobacco use, readiness to quit, quit attempts, and use of smoking cessation services. In addition, we looked at the anticipatory phase to study changes in readiness to quit that could inform interventions and optimize the effect of a tax increase among groups most likely to have a shift in readiness to quit. The purpose of this study was to assess the use of an EHR to monitor the effect of federal tobacco control policy change on the low-income patients served by the Tobacco Control Initiative (TCI) smoking cessation service in the public hospital system.

## Methods

The TCI was established in partnership with the Louisiana State University Health Care Services Division (LSU HCSD) to address the needs of low-income tobacco users in Louisiana. The TCI, using the recommendations of the Clinical Practice Guideline (17), provides cessation services in the state public hospital system. These cessation services include implementing a system for identifying tobacco users, providing staff-coordinated services to tobacco users, developing and promoting policies supporting treatment, reducing of out-of-pocket expenses for patients receiving treatments, and promoting the 5 A’s (17) for addressing tobacco use. In addition, the TCI provides self-help material for those desiring to quit on their own, cessation medication for uninsured and financially indigent patients, free sessions of group behavioral counseling, and access to free telephone counseling via the Louisiana quitline.

The LSU HCSD is the largest provider of health care to Louisiana's uninsured citizens and serves a large clientele of low-income patients (18). Approximately 60% of adult patients in the LSU HCSD outpatient clinic population are uninsured; most of those patients (45% of all adult clinic patients) are eligible for free care under Louisiana law. Free care eligibility is determined by household income and household size, with eligibility available to patients from households falling below 200% of the federal poverty level. The TCI was implemented in 7 of 10 facilities of the LSU public hospital system, providing health care throughout the state in one of the largest safety-net organizations in the nation. The LSU Health Shreveport system, representing 3 of the 10 public hospitals, were not available in the system. 

At the time of this study, the LSU HCSD used an internally developed electronic data repository called CLIQ (for clinical inquiry), a Web-based, result-reporting application with a clinical user interface. CLIQ, which provides clinicians an entry point to patient data, maintains 16 real-time data interfaces from clinical and administrative feeder systems (18). The smoking information was collected by the CLIQ system starting December 2008. In 2009, the screening rate was 76% to 79% and the rate of assessing readiness to quit was 82% to 87%.

Outpatient clinic visits of persons 18 years or older to the 7 LSU facilities were followed from February 1, 2009, through May 31, 2009. Clinic staffs were prompted in the EHR to assess patients’ smoking status and readiness to quit tobacco use. Smoking status was defined based on answers to the question, “Patient used tobacco in the past 30 days?” Readiness to quit was assessed based on the question, “Patient ready to quit in next 30 days?”

The distributions of readiness to quit smoking in the next 30 days in different subgroups defined by demographic characteristics are presented. Differences in readiness to quit across demographic groups were evaluated using the χ^2^ contingency table tests. To compare readiness to quit before and after April 1, 2009, generalized estimating equations model (GEE) was conducted using SAS 9.1 (SAS Institute Inc, Cary, North Carolina). In our data set, some patients had measurements from more than 1 visit. For taking the within-person correlations into consideration, we revised our analyses using the GEE with an auto-regressive correlation structure.

## Results

**Figure 1:**
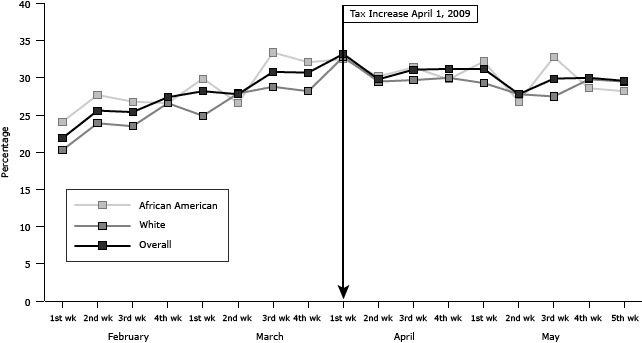
Weekly rate of readiness to quit tobacco use in next 30 days by race, Louisiana, February through May 2009. A federal cigarette tax increase of $0.61 per pack went into effect April 1, 2009. **Table:** 

From February 1, 2009, through May 31, 2009, there were 95,807 clinic visits by 34,828 (36%) men and 60,979 (64%) women. Half of the participants (50%) were aged 50 or older; 20% were self-pay or had commercial insurance. Of these men and women, 31% were smokers. Smokers who were female, aged 31 or older, African American, self-payers, and assessed at a clinic visit in April were most likely to report that they were ready to quit smoking within the next 30 days (Table 1). Overall, the rate of readiness to quit increased from 24.9% in February to 31.4% in April. For all smokers, the rate of readiness to quit increased from 22% during the first week of February to 33% during the first week of April (Figure 1). After the tax increase went into effect, however, the proportion of those ready to quit decreased to 29% by the fifth week of May.

**Table 1:** Readiness to Quit Tobacco Use Within the Next 30 Days by Demographic Status, Louisiana, February through May 2009

Variable	Frequency	%	*P* a
**Sex**			
Female	5,056/16,348	30.9	<.001
Male	3,485/12,945	26.9	
**Age, y**			
≤30	1,104/4,246	26.0	<.001
31–40	1,268/4,191	30.3	
41–50	2,592/8,750	29.6	
51–60	2,538/8,462	30.0	
≥61	1,025/3,572	28.7	
**Race**			
White	4,107/14,718	27.9	<.01
African American	3,968/13,248	30.0	
**Payer**			
Commercial insurance	499/2,232	22.4	<.001
Free care or indigent	4,237/14,231	29.8	
Medicaid	1,394/4,635	30.1	
Medicare	810/3,059	26.5	
Self-pay	1,240/3,676	33.7	
**Time**			
February	1,378/5,528	24.9	<.001
March	2,366/7,961	29.7	
April	2,426/7,737	31.4	
May	2,371/8,067	29.4	

a χ^2^ test.

Race	February				March				April				May				
	1st wk	2nd wk	3rd wk	4th wk	1st wk	2nd wk	3rd wk	4th wk	1st wk	2nd wk	3rd wk	4th wk	1st wk	2nd wk	3rd wk	4th wk	5th wk
Overall	21.9	25.6	25.4	27.4	28.2	27.8	30.8	30.7	33.2	29.8	31.1	31.2	31.2	27.8	29.9	30.0	29.6
White	20.3	23.9	23.5	26.6	24.9	27.9	28.8	28.2	32.8	29.5	29.7	30.0	29.3	27.8	27.5	29.8	29.5
African American	24.1	27.7	26.8	26.6	29.9	26.6	33.4	32.1	32.5	30.2	31.4	29.8	32.2	26.8	32.8	28.6	28.2

For African American and white smokers, changes in readiness to quit were similar (Figure 1). For both groups, the rates of readiness to quit increased by about 50% as the effective date of the federal tax increase approached. African American smokers responded more immediately to the anticipated price increase than white smokers. For African Americans, the rate of readiness to quit increased from 24% in the first week of February to 33% in the third week of March and then decreased to 28% in May. For whites, readiness to quit increased from 20% in the first week of February to 33% in the first week of April but decreased to 30% at the end of May.

**Figure 2:**
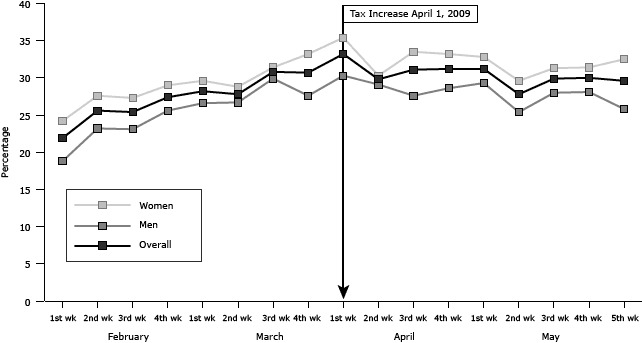
Weekly rate of readiness to quit tobacco use in next 30 days by sex, Louisiana, February through May 2009. A federal cigarette tax increase of $0.61 per pack went into effect April 1, 2009. **Table:** 

Trends in changes in readiness to quit between male and female smokers were similar (Figure 2). Women more often reported being ready to quit within the next 30 days than men. Changes in readiness to quit by age group varied. Those who were aged 30 years or younger generally had lower rates of readiness to quit than those older than 30 years.

Sex	February				March				April				May				
	1st wk	2nd wk	3rd wk	4th wk	1st wk	2nd wk	3rd wk	4th wk	1st wk	2nd wk	3rd wk	4th wk	1st wk	2nd wk	3rd wk	4th wk	5th wk
Overall	21.9	25.6	25.4	27.4	28.2	27.8	30.8	30.7	33.2	29.8	31.1	31.2	31.2	27.8	29.9	30.0	29.6
Men	18.8	23.2	23.1	25.6	26.6	26.7	29.9	27.6	30.3	29.1	27.6	28.6	29.3	25.4	28.0	28.1	25.8
Women	24.2	27.6	27.3	29.0	29.6	28.8	31.4	33.2	35.4	30.3	33.5	33.2	32.8	29.6	31.3	31.4	32.5

Table 2 presents the results of GEE model for readiness to quit in the next 30 days by 3 periods including the full 4-month period (full regression model, February–May), the 2 months before implementation of the tax (February–March), and the 2 months after the tax increase (April–May). For the full regression model (February–May), smokers who were assessed at a clinic visit in April were more likely to be ready to quit than those assessed in February (OR, 1.39; 95% CI, 1.28–1.51) after adjusting for demographic factors and payer status. To compare readiness to quit before and after the April 1 tax increase, 2 models were analyzed. For both periods, February through March and April through May, smokers who were ready to quit were more likely to be female, to self-pay for their health care compared with those who had commercial insurance, and to be aged 31 to 40 years compared with those aged 30 years or younger (Table 2). African Americans were more likely than whites to report readiness to quit in February through March (OR, 1.17; 95% CI, 1.09–1.28). However, the difference was not significant (OR, 1.06; 95% CI, 0.98–1.15) in April through May (Table 2). For both periods, smokers who self-pay were more likely to report readiness to quit within 30 days than those with commercial insurance (OR, 1.8; 95% CI, 1.50–2.19 and OR, 1.72; 95% CI, 1.43–2.05).

**Table 2:** Generalized Estimating Equations Model for Readiness to Quit Tobacco Use Within the Next 30 Days, Louisiana, February through May 2009

Variable	February–May		February–March		April–May	
	OR (95% CI)	*P*	OR (95% CI)	*P*	OR (95% CI)	*P*
**Sex**						
Female	1 [Reference]		1 [Reference]		1 [Reference]	
Male	0.85 (0.80–0.90)	<.001	0.85 (0.78–0.93)	<.001	0.84 (0.78–0.91)	<.001
**Age, y**						
≤30	1 [Reference]		1 [Reference]		1 [Reference]	
31–40	1.23 (1.11–1.37)	<.001	1.15 (0.99–1.35)	.08	1.29 (1.12–1.49)	<.001
41–50	1.21 (1.10–1.33)	<.001	1.13 (0.99–1.29)	.08	1.27 (1.12–1.44)	<.001
51–60	1.22 (1.11–1.34)	<.001	1.14 (0.99–1.30)	<.06	1.27 (1.12–1.44)	<.001
≥61	1.19 (1.06–1.34)	.004	1.10 (0.94–1.30)	.29	1.25 (1.07–1.47)	<.001
**Race**						
White	1 [Reference]		1 [Reference]		1 [Reference]	
African American	1.11 (1.04–1.18)	<.001	1.17 (1.09–1.28)	<.001	1.06 (0.98–1.15)	.13
**Payer**						
Commercial insurance	1 [Reference]		1 [Reference]		1 [Reference]	
Free care or indigent	1.45 (1.29–1.63)	<.001	1.41 (1.19–1.66)	<.001	1.48 (1.26–1.73)	<.001
Medicaid	1.47 (1.29–1.67)	<.001	1.49 (1.24–1.79)	<.001	1.42 (1.19–1.69)	<.001
Medicare	1.24 (1.07–1.43)	.004	1.10 (0.89–1.36)	.37	1.36 (1.12–1.66)	.002
Self-pay	1.76 (1.54–2.01)	<.001	1.81 (1.50–2.19)	<.001	1.72 (1.43–2.05)	<.001
**Time**						
February	1 [Reference]		NA			
March	1.28 (1.18–1.39)	<.001				
April	1.39 (1.28–1.51)	<.001				
May	1.24 (1.14–1.35)	<.001				

Abbreviations: OR, odds ratio; CI, confidence interval; NA not applicable.

## Discussion

This study demonstrates the effect of a substantial tax increase on readiness to quit among low-income smokers. Our results show that a cigarette tax increase offers an opportunity to intervene with smokers who are ready to quit. These conclusions agree with previous reports showing that smokers are more likely to try to quit when the price of cigarettes increases (5,7,19). The high tobacco use among low-income groups is reflected in their disproportionate burden of illnesses attributable to smoking (20), and many of these smokers express interest in quitting (19,21). Farrelly et al showed that low-income smokers spent a larger share of their income on cigarettes than higher-income families in 2011 (22). Nationally, those with the lowest incomes spend about 14.2% of their income on cigarettes compared with smokers in the highest income group who spent 2%. These findings show the need to mitigate the health and economic aspects of tobacco use.

Primary care visits offer an opportunity to enhance the combined effect of evidence-based treatment, policy change such as quitline counseling, pharmacotherapy, and tax increases. Readiness to quit may result from the smoking cessation campaign or the combination of the campaign and tax increases. However, we consulted via e-mail the program directors for the Louisiana Department of Health and Hospitals’ tobacco control program and Tobacco-Free Living, a statewide tobacco control program funded by state excise tax and searched the Centers for Disease Control and Prevention’s website and found that none of the organizations had implemented a mass reach health communication intervention related to the federal tax increase. The finding of a decrease in readiness to quit after the tax went into effect is similar to findings from a study that showed that the number of monthly calls to the quitline more than doubled before a tax increase; afterwards, the number decreased slowly (23). Although the tax increase may reduce the consumption of cigarettes, the tobacco industry may react to this reduction with marketing approaches such as reducing prices at the point of sale (24).

Most studies show that when the price of cigarettes increases, demand decreases (5). Increases in price reduce the purchasing power of smokers, and, hence, their consumption of cigarettes. An increase in the tobacco tax decreases the smoking rate among younger and lower-income populations (19). Further, we found that smokers who self-pay for health care and those with Medicaid support are more likely to report readiness to quit than smokers who have commercial insurance. At the time of the study, commercial insurance in Louisiana did not cover cessation benefits. People without cessation benefits who paid for them might have an increased likelihood of being ready to quit. Hence, the observed increase in readiness to quit may be due to financial benefit from insurance or financial stress (25).

Limitations to the results include use of self-reported data, the unavailability of data, the cross-sectional design, and limited sampling. In this study, the CLIQ data collection system minimized the odds of recall bias or false reporting from patients and clinicians. Some variables such as education level, level of addiction, and some psychosocial variables that may be potential confounders were not included in this study because these variables are not available in the EHR system. The cross-sectional design limits conclusions about the causal relationship of risk factors and readiness to quit. This study did not have a control group to compare findings with another state or with outpatients within the state.

A notable feature of our study is that it involved a health informatics system providing real-time collection of data to evaluate the influence of a cigarette tax increase and to identify trends of readiness to quit among lower-income people. The use of EHR provides clinicians with immediate information related to risk factors and tracks smoking patterns for patients (26). This study demonstrates that an EHR can be used to monitor the effect of federal tobacco control policy change in the public hospital system. McCullough et al found that physician-documented counseling rates are significantly higher when patients are asked smoking-related questions as part of an electronic medical records system (27). This is a strategy to improve smoking cessation counseling in clinical settings.

The Patient Protection and Affordable Care Act (28) is expected to lead to an increase in the use of EHRs in health care delivery systems. This is an opportunity to use comparable data sets with a minimum standard for inclusion of tobacco use in EHRs to ensure that the data can be used in a meaningful way. Because cessation interventions may act synergistically with cigarette price increases, future studies should identify the profiles of smokers, especially among lower-income people, who are ready to quit. For example, the combination of antismoking programs and increased tobacco taxes reduced the level of cigarette consumption among youths more than expected as a result of price increases alone (9,29). In the future, analyses involving multiple factors, such as daily smoking amounts, menthol cigarette use, withdrawal status, family income, previous cessation experience, and a more comprehensive study design, may be applied.

A health informatics system or electronic health records system that efficiently tracks trends in readiness to quit can be used in combination with other strategies and thus optimize efforts to control tobacco use. For instance, EHR with interactive voice response can optimize or improve adherence to or use of smoking cessation services. Our data suggest that a cigarette tax increase affects smokers’ readiness to quit and provides an opportunity to intervene at the most beneficial time. Readiness to quit peaked in April and then declined after the tax increase came into effect. The anticipatory phase may be a viable opportunity for smoking cessation interventions among this population. We also found that reactions of low-income African American smokers to the tax increase occurred before that of whites. An understanding of the effect of tax increases on readiness to quit can provide a foundation for effective intervention. With a health informatics system, smoking cessation interventions using multiple strategies including a federal cigarette tax increase and cessation services like the TCI within a health care delivery system can work synergistically to enhance cessation across populations of smokers.
